# Bufei Qingyu Granules Inhibit the Development of Systemic Sclerosis via Notch-1/Jagged-2 Signaling Pathway

**DOI:** 10.1155/2019/6709278

**Published:** 2019-07-02

**Authors:** Minhui Su, Fang Tian, Bingchen Ouyang, Xiaoyu Wu, Feng Guo, Hua Chen, Xuejun Zhu, Jianmei Chen, Xian Qian

**Affiliations:** ^1^Affiliated Hospital of Nanjing University of Chinese Medicine, Nanjing, Jiangsu 210029, China; ^2^Department of Center Laboratory, Jiangsu Province Hospital of Traditional Chinese Medicine, Nanjing, Jiangsu 210029, China; ^3^Department of Clinical Pharmacology, Affiliated Hospital of Nanjing University of Chinese Medicine, Nanjing, Jiangsu 210029, China; ^4^State Key Laboratory of Natural Medicines, Jiangsu Key Laboratory of Drug Screening, School of Life Science and Technology, China Pharmaceutical University, 24 Tongjia Xiang, Nanjing, Jiangsu 210009, China; ^5^Department of Rheumatology, Jiangsu Province Hospital of Traditional Chinese Medicine, Affiliated Hospital of Nanjing University of Chinese Medicine, Nanjing, Jiangsu 210029, China; ^6^Nursing College, Nanjing University of Chinese Medicine, Nanjing, Jiangsu 210029, China

## Abstract

Systemic sclerosis (SSc) is a rare chronic autoimmune disorder, mainly characterized by skin sclerosis. In this study, Bufei Qingyu Granules (BQG), a Chinese herbal formula, was used to treat SSc. To better understand the effects and molecular mechanisms of BQG, we successfully established a Bleomycin- (BLM-) induced SSc mouse model, and the mice were treated by BQG. Meanwhile, transcriptomic and bioinformatics analyses were conducted on those samples. As a result, we visually showed that BQG ameliorated the overall health of mice, including body weight, spleen, and thymus index. Thus, it also significantly alleviated inflammation presented by Chemokine (C-X-C motif) ligand 2 (Cxcl2), vasculopathy characterized by *α*-smooth muscle actin (*α*-SMA), and fibrotic changes elaborated by not only pathological images, but also the hydroxyproline (HYP) content. After testing by transcriptomic analysis, Cxcl2, Synaptosomal-associated protein 25 (Snap25), and Eukaryotic translation initiation factor 3, and subunit J2 (Eif3j2) which were differentially expressed genes, were verified, so that the data were credible. We further found that BQG could regulate Notch signaling pathway by significantly decreasing both mRNA and protein expression levels of Notch-1 and Jagged-2. Hence, this study demonstrated that BQG could ameliorate the sclerotic skin in mice model involved in inflammation, vascular changes, and fibrosis effects, which was partly mediated by Notch signaling pathway.

## 1. Introduction

Systemic sclerosis (SSc) is a rare chronic autoimmune rheumatic disease characterized by persistent extensive fibroproliferation of skin and multiple visceral organs [1]. According to the severity and the involved area, the localized scleroderma should be differentiated from the limited cutaneous scleroderma [2]. Although pathogenesis and the underlying molecular mechanism should be fully elucidated, there is no doubt that this can be attributed to three backbones as follows: innate immune, vasculopathy, and fibrosis [3–5].

Although treatment of SSc has greatly attracted the scholar' attention, there is currently no curative therapy resistant to the mentioned disorder [6]. In Chinese medicine, as supplement of modern medicine, there is a treasure, which need to be exploited, especially for this complex disease [7–9]. Bufei Qingyu Granules (BQG), that is a traditional Chinese medicine (TCM), is composed of* Astragalus mongholicus* (Huangqi),* Salvia miltiorrhiza* (Danshen),* Angelica sinensis* (Danggui), and other seven Chinese herbs on the basis of a certain proportion of composition.* Astragalus mongholicus* and* Angelica sinensis* mixture may have antifibrotic effects on renal tubulointerstitial fibrosis and nephrotic syndrome [10, 11]. Compound* Astragalus* and* Salvia miltiorrhiza* extractions would be also beneficial for fibrotic diseases, such as liver fibrosis and hypertrophic scar [12–14]. BQG has been developed and extensively used by Jiangsu Province Hospital of TCM (Nanjing, China) for the treatment of SSc and a portion of pulmonary fibrosis, demonstrating to be highly beneficial in clinical practice. In a preliminary evidence, we previously found the utility of alleviating sclerotic skin with appropriate dosage of BQG in Bleomycin- (BLM-) induced mice model [15].

In recent years, transcriptomics and bioinformatics analyses, providing important lines for understanding of genes' regulations and the mechanisms behind them, were extensively applied in TCM-based researches [16, 17]. Here, in order to deeply explore the treatment efficacy of BQG, we obtained a relatively reliable material and sample basis, and the high-throughput sequencing and bioinformatics approaches were subsequently carried out to find out differentially expressed genes (DEGs) and analyze the effects of BQG at both gene and protein expression levels. In addition, we aimed to reveal the potential targets and signaling pathways associated with the treatment efficacy of BQG for SSc.

## 2. Materials and Methods

### 2.1. Ethics Statement

All animal experiments were strictly performed in accordance with the “Guide for the Care and Use of Laboratory Animals” published by the National Institutes of Health (NIH; Bethesda, MD, USA). This study was reviewed and approved by the Ethics Committee of Nanjing Medical University (Nanjing, China).

### 2.2. Preparation of BQG Samples

A total of 10 individual BQG components were commercially available in form of solid granules provided by JiangYin TianJiang Pharmaceuticals Co. Ltd. (Jiangsu, China), which were packaged in 0.5-3 g per bag for each herb. The detailed information of each herb granule was presented in Table 1. Based on our preexperiments, all the herb granules were dissolved in distilled water as solvent to make 1.736 g/mL BQG by an ultrasonic bath for 1 h and then stored at 4°C. In addition, BQG was fully oscillated and mixed before treating mice.

BQG was quantitated and mixed with double volume of pure methanol, and then the solution was under vortex for 1 min and centrifuged at 12,000 rpm for 10 min at 4°C. The supernatant was filtered through a membrane filter (0.45 *μ*m). Gradient dilution of the filtrate was conducted with 50% methanol before ultra-high-performance liquid chromatography combined with quadrupole-time-of-flight mass spectrometry (UHPLC-Q-TOF/MS) analysis.

### 2.3. UHPLC-Q-TOF/MS Analysis

This analysis was carried out by using an Agilent 1290 Infinity LC system (Agilent Technologies, Santa Clara, CA, USA) in combination with a Triple TOF 5600 system equipped with an electrospray ionization (ESI) source (AB SCIEX, Framingham, MA, USA). An XTerra® MS C18 column (2.1 mm × 100 mm, 3.5 *μ*m) was used for the purpose of chromatographic separation and its temperature was maintained at 40°C during the analysis. The mobile phase was composed of 0.1% aqueous formic acid (A) and acetonitrile (B). The gradient elution program could be used for treatment with 5% B for 0-2 min, with 5%-90% B for 2-9 min, and with 90% B for 9-11 min, and posttreatment for 12-14 min. The flow rate was 200 *μ*L/min and the injection volume was 10 *μ*L. Positive and negative ion modes were operated, respectively, for the analysis. An ESI source was applied with parameters as follows: Ion Spray voltage, 4500 V; ion source gas 1 (N2), 50 Arb; ion source gas 2 (N2), 50 Arb; gas temperature, 550°C; curtain gas, 35 Arb; declustering potential, 80 V. The mass spectrum was acquired from 60 to 1500 m/z, and the collision energy was 10 eV.

### 2.4. Mice

Female BALB/c mice with the age of 6-week-old were purchased from the Experimental Animal Center of Nantong University (Nantong, China) and were maintained in specific pathogen-free mouse colonies with a 12 h light cycle and temperature varying between 25 and 28°C. Relative humidity was maintained between 50% and 60%. Before the start of study, all the mice were acclimated to laboratory conditions for within one week. Then, all the mice were randomly divided into three groups as follows: control group (n=6); BLM group (n=6); BLM + 34.72 g/kg BQG treatment group (n=6, dosage was selected according to a previous study [15]).

### 2.5. Mouse Model of SSc and BQG Treatment

Here, BLM (Hisun-Pfizer Pharmaceuticals Co. Ltd., China) was dissolved in phosphate-buffered saline (PBS) at the concentration of 400 *μ*g/mL and sterilized by filtration as well. Mice underwent a subcutaneous and daily injection of 100 *μ*L BLM or PBS solution into a single location on the shaved backs of mice with a 0.45 mm needle. The injection was carried out successively for 4 weeks as previously described [18]. Drug intervention was started during BLM or PBS subcutaneous injection. In the case of BLM exposure model, mice were treated with 0.2 mL/10 g BQG via oral gavage. In addition to BLM group and control group, normal vehicle (PBS) was administered with an equal volume in the same manner.

Mice were weighted and sacrificed 24 h after the last dosage. Some organs and the shaved back of skins were resected for further studies after quick freezing in liquid nitrogen, which were then preserved at -80°C.

### 2.6. Overall Health Assessment

Before mice to be sacrificed, the condition of mice and their skin were daily observed. Body weight (g) of mice was measured and noted weekly. After mice were sacrificed, the lung, spleen, and thymus were taken out and index of organs was calculated in each group of mice according to the following formula: index of organ = weight of organ (mg) / body weight (g) *∗* 100%.

### 2.7. Histopathological Examination

All skin sections were cut from the paramidline, lower back shaved region. The skin pieces which were fixed in 4% paraformaldehyde for 24 h were embedded in paraffin routinely. A 5 *μ*m-thick tissue section was stained with hematoxylin and eosin (H&E) and Masson's trichrome stain. We evaluated dermal thickness, which was defined as the thickness of skin from the dermal-epidermal junction to the junction between the dermis and subcutaneous fat [19, 20]. The thickness of dermis was calculated from six different randomly selected fields per specimen by using Image J software (NIH, Bethesda, MD, USA). Slides were examined by standard bright-field microscopy (Nikon Ni-U, China) by two pathologists who were single blinded to the experimental group assignment.

### 2.8. Determination of Hydroxyproline Content in Skin Tissue

Hydroxyproline (HYP) assay was used to measure collagen contents. Following manufacturer's instructions of HYP-detection kits (Nanjing Jiancheng Biological Engineering Research Institute, Nanjing, China), 6 mm punch biopsy specimens of shaved back skin tissues were hydrolyzed. The supernatants were collected after chain reaction, and the HYP content was quantified by colorimetric analysis at 550 nm (Synergy HT Microplate Reader; BioTek Instruments, Inc., Winooski, VT, USA) in mice of each group.

### 2.9. Enzyme-Linked Immunosorbent Assay (ELISA)

Chemokine (C-X-C motif) ligand 2 (Cxcl2) levels in mice serum were collected by cardiac puncture before to be sacrificed and were assessed using ELISA kits (Nanjing Jin Yibai Biological Technology, Nanjing, China) in accordance with the manufacturers' instructions. The final results were presented in histogram.

### 2.10. Immunostaining

Immunohistochemistry was carried out using antibodies directed against *α*-smooth muscle actin (Proteintech, Rosemont, IL, USA) abiding by the routine protocols as previously described [21]. Then, all slices were examined independently by two investigators in a blinded manner.

### 2.11. Total RNA Extraction

Total RNA isolation from skin tissues was executed respectively by using the TRIzol reagent (Invitrogen, Carlsbad, CA, USA) in each group according to the manufacturer's introductions. The quantity and purity of RNA were measured by using Qubit 2.0 Fluorometer (Thermo Fisher Scientific, Waltham, MA, USA); thus, RNA integrity was verified by agarose gel.

### 2.12. Transcriptomic Assay and Bioinformatics Analysis

High-quality RNA was used for library construction and high-throughput sequencing. RNA sequencing library was carried out using the VAHTSTM mRNA-seq V2 Library prep Kit (Illumina, Chicago, IL, USA) according to the manufacturer's protocols. The library was then sequenced on a Hiseq platform (Illumina, Chicago, IL, USA) by Sangon Biotech Co., Ltd. (Shanghai, China).

Transcriptome analysis was undertaken using mice's reference genome-based reads mapping. Gene expression levels were estimated using Transcripts Per Million (TPM) values. High-throughput sequencing was performed through applying the criteria of |log_2_ fold change|>1,* P*-values<0.05, and at least one group's mean TPM≥5 as DEGs, which was employed for subsequent analysis.

### 2.13. Quantitative Reverse Transcription Polymerase Chain Reaction (RT-qPCR)

The process of RNA extraction was carried out as mentioned previously. Here, 1 *μ*g of total mRNA was reverse-transcribed into cDNA synthesized with HiScript® II One Step RT-qPCR Probe Kit (Illumina, Chicago, IL, USA). Target genes were analyzed by RT-qPCR according to the manufacturer's instructions (Applied Biosystems Inc., Foster City, CA, USA). The associated primer sequences are listed in Table 2. The relative mRNA expression levels were quantified with the 2^−ΔΔCt^ method and the amplified transcript level of each specific gene was normalized to the expression of the endogenous control GAPDH.

### 2.14. Antibodies and Western Blot Analysis

The radio immunoprecipitation assay (RIPA) lysis buffer (Solarbio, Beijing, China) was added into skin tissues, while the tube was placed on ice for 40 min. The tissue was then centrifuged at 12000 rpm for 5 min at 4°C to remove pellet. The lysate, which was transferred to a fresh tube, was measured by using BCA Protein Assay kit (Thermo Fisher Scientific, Waltham, MA, USA). Add 5 × SDS stop buffer to the lysate so as to reach 1 × SDS final. Load denatured mixture of protein and protein markers into the gel and transfer onto Polyvinylidene difluoride (PVDF) membranes. After electrotransferring and blocking with 1 × TBST containing 5% nonfat dry milk, membranes were incubated with primary antibodies overnight at 4°C: mouse monoclonal GAPDH (1:10000; Proteintech, Rosemont, IL, USA), rabbit monoclonal Anti-Jagged2 (1:500; Abcam, Cambridge, UK), and Notch1 XP®rabbit mAb (1:500, Cell Signaling Technology, Danvers, MA, USA). Then, the membranes were incubated with the horseradish peroxidase- (HRP-) conjugated secondary antibody diluted at 1:2000 (Proteintech, Rosemont, IL, USA) at room temperature for 1 h. Last but not least is the exposure under a chemiluminescence imaging system (Bio-Rad Laboratories, Hercules, CA, USA). GAPDH was used as a loading control. All the protein bands were analyzed by using Image J software.

### 2.15. Statistical Analysis

The data were presented as mean ± standard deviation (SD). Data analysis was performed by using GraphPad Prism 6 software, version 6.0a (GraphPad Software, USA). Averaged data of two groups were compared using unpaired two-sample t-test. One-way analysis of variance (ANOVA) with Bonferroni adjustment were used to perform multiple group comparisons. A* P*-value<0.05 was considered statistically significant.

## 3. Results

### 3.1. Typical Chemical Components and Their Contents in BQG Are Identified by UHPLC-Q-TOF/MS

**Figure 1:**
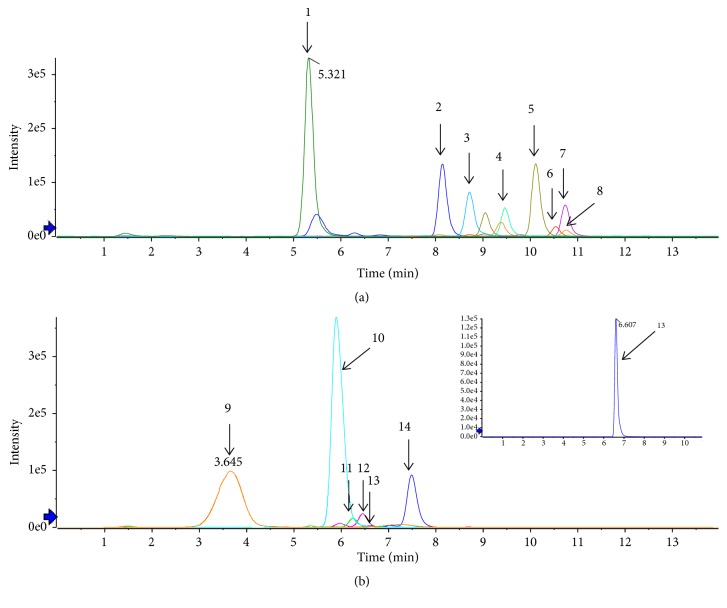
Fingerprints of BQG samples in the positive (a) and negative (b) ion modes by UHPLC-Q-TOF/MS. Peak 1, Amygdalin; Peak 2, Paeonol; Peak 3, Schizandrol A; Peak 4, Dihydrotanshinone I; Peak 5, Cryptotanshinone; Peak 6, Schizandrin A; Peak 7, Tanshinone IIA; Peak 8, Schizandrin B; Peak 9, Danshensu; Peak 10, Acteoside; Peak 11, Ferulic acid; Peak 12, Lobetyolin; Peak 13, Platycodin D; Peak 14, Astragaloside IV.

To clarify the material basis of BQG for the treatment efficacy, the representative chemical components in BQG confirmed by UHPLC-Q-TOF/MS were detected under the optimized conditions. As data are shown in Figures 1(a) and 1(b) and Table 3, a total of 14 peak signals and the content of each constituent were identified and calculated, respectively.

### 3.2. Effects of BQG Are Showed in Overall Health of Mice

**Figure 2:**
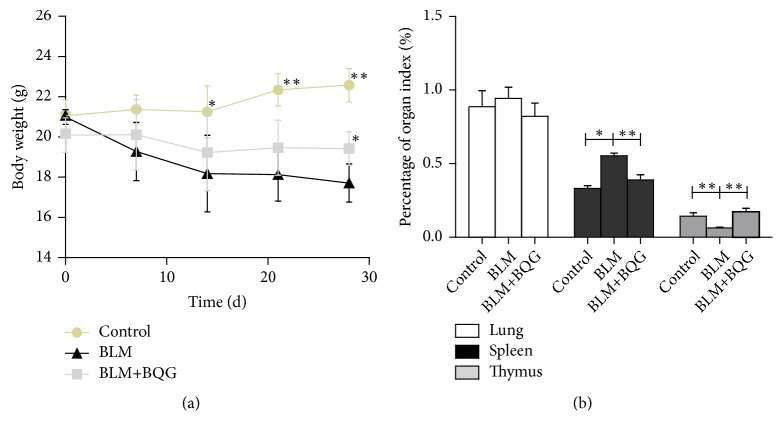
Overall health assessment after BQG treatment in SSc mouse model. (a) The curves of body weight of mice in different groups at different time points. (b) The percentage of lung, spleen, and thymus index, n=6 per group; ^∗^*P*<0.05, ^∗∗^*P*<0.01 compared with the model group.

In this experiment, the overall health of mice was observed as a routine. The conditions of mice in the control group treated with PBS, which included the spirit, food intake, activity, and body weight, were much better than the BLM group at the end of the point, and also the hair in shaved area was almost recovered. Compared with the model group, the status of BQG-treated mice exceeded; thus, the degree of dermal sclerosis was lighter, not as the hair around the injection site stopping growing. There was no significant difference in body weight of mice between each group before the start of the experiment (*P*=0.056). The mice in model group had the lowest body weight among the three groups. BQG-treated group had the higher body weight compared with the model group (19.4983 ± 0.83968 g vs 17.7833 ± 0.94534 g,* P*=0.004; Figure 2(a)). The organ index, including lung, spleen, and thymus, was used to reflect the status of the animal's function in a chronic drug experiment, especially for the thymus and spleen as important indicator reflecting the immune function and splenomegaly occurrence of the body to some extent. As illustrated in Figure 2(b), there was no significant difference in lung index between the groups,* P*>0.05. There was apparently lower level of spleen index in BQG-treated group compared with the model group (*P*<0.001). However, the level of thymus index obviously increased compared with the model group (*P*<0.001).

### 3.3. BQG Attenuates Skin Sclerosis in BLM-Induced Animal Model

**Figure 3:**
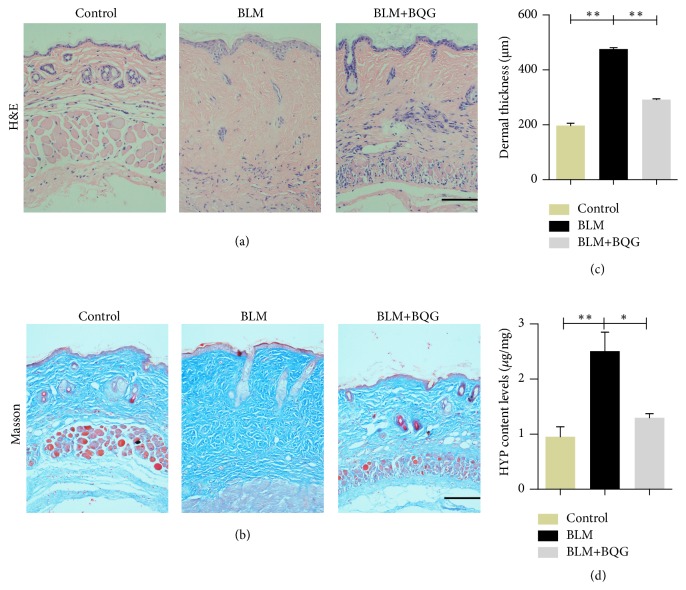
Effects of BQG in improving BLM-induced skin sclerosis of mouse model. Skin sections derived from BLM or PBS-treated mice in the presence or absence of BQG administration were stained with hematoxylin and eosin (a) and Masson's trichrome (b). Representative images are shown. Scale bar=100 *μ*m. (c) Data of dermal thickness are presented by histogram. (d) The levels of HYP content in each group of mice.

In this section, our eyes were fixed to indicate whether BQG has the valid effects on BLM-induced mice model. To the end, lesional skin sites were thicker in BLM-induced mice than in PBS-treated mice on 28th day, and no effects were observed on the shaved skin of PBS-treated mice. However, dermal thickness was visualized to be obviously decreased by BQG intervention in both slices stained by H&E and Masson's trichrome (Figures 3(a) and 3(b)). Moreover, histogram was used to quantify dermal thickness and levels of HYP content in each group of mice, which showed that both of two items were significantly reduced in BLM exposure mice treated with BQG compared with those which did not receive BQG (Figures 3(c) and 3(d)).

**Figure 4:**
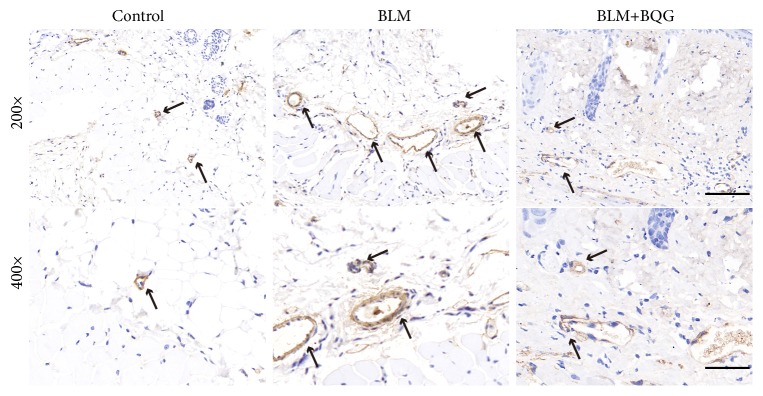
*α*-SMA expression was evaluated by immunohistochemistry. Black arrows indicated *α*-SMA-positive cells in vascular walls. Representative images are shown. Original magnifications are 200× (scale bar=100 *μ*m) and 400× (scale bar=50 *μ*m), respectively.

**Figure 5:**
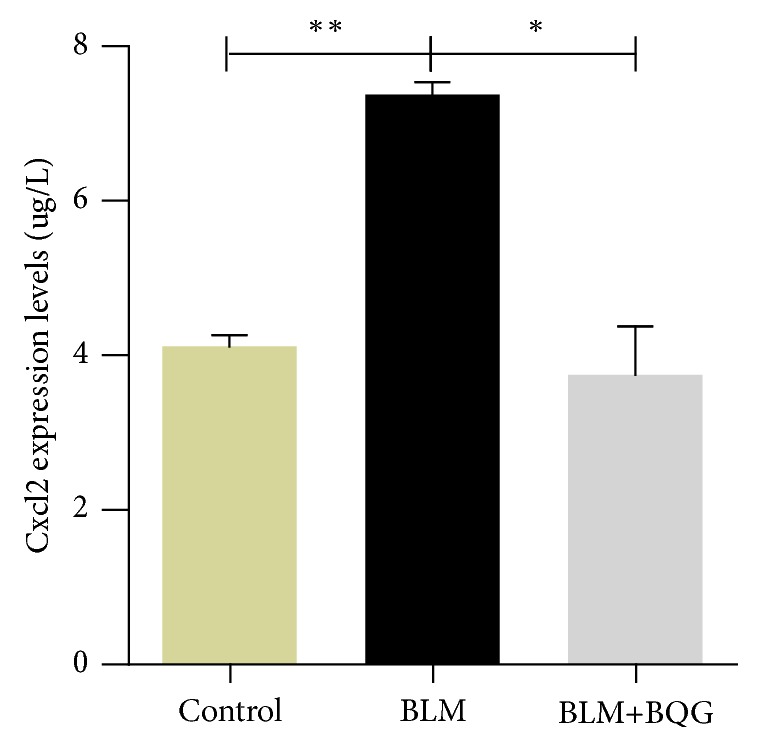
ELISA assay showed elevated Cxcl2 in the serum derived from mice in different groups. Each graph illustrates mean ± SD of the indicated parameters (n≥3 per group; ^∗^*P*<0.05, ^∗∗^*P*<0.01 vs BLM model group).

In addition, *α*-smooth muscle actin (*α*-SMA) expression was evaluated by immunohistochemistry, which showed that proliferation of positive vascular smooth muscle cells visualized as like vessel wall thickness was decreased in BQG group, compared with the BLM group mice (Figure 4). Besides, inflammation involved in this progress was detected by the production levels of Cxcl2. As shown in Figure 5, BQG group cut the expression by almost half percent compared with BLM model group.

Taken together, these results indicated that BQG had a potential inhibition role in inflammation, vascular changes, and fibrosis in the BLM-induced SSc mouse model.

### 3.4. DEGs Are Expressed between BLM Model and BQG-Treated Group

**Figure 6:**
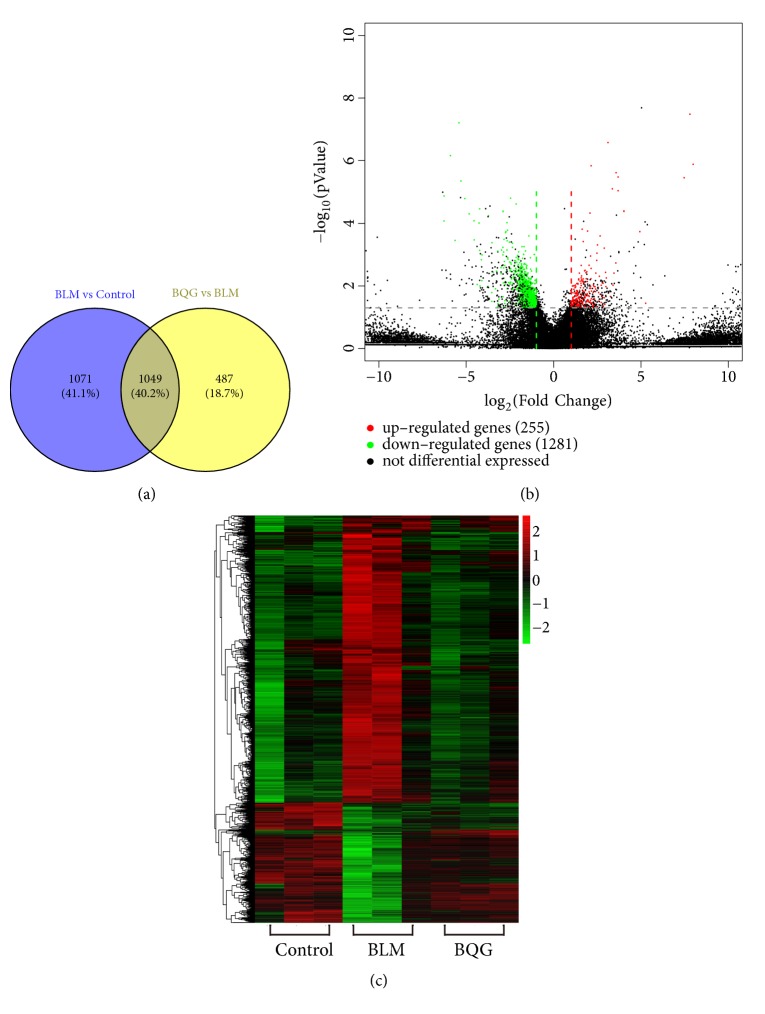
Transcriptomic assay and bioinformatics analysis. (a) Venn diagram of overlapping genes derived from transcriptome analysis in a pairwise comparison. (b) Compared with BLM group, filtering of significant DEGs was presented by valcano. (c) Heat map of regulated DEGs in skin samples of control, BLM, and BQG-treated groups. Red color represents upregulated genes, while green color shows downregulated genes. The depth of color represents difference on multiplier.

A comparative analysis between the control, BLM, and BQG-treated groups for transcriptome analysis and gene expression was conducted. In total, the RNA-Seq results included within 51826 genes. As displayed in Figure 6(a), both up- and downregulated DEGs were overlapped. Of these, transcriptomic analysis revealed that, compared with the control group, 1502 genes were upregulated in the BLM model group, among which 945 genes were changed in the BQG-treated group. In addition, 618 genes were downregulated in the BLM model group, among which 103 genes were reversed in the BQG-treated group. From Figures 6(b) and 6(c), the condition of DEGs, especially between the BLM model and BQG treatment groups, could be observed. By the way, the pathologic changes of sample 3 were not as severe as that another two. Maybe this is the reason why sample 3 of BLM group is a little bit different from another two in heat map. Relatively small sample size may also be a factor to lead to this phenomenon. Thus, adding sample 3 will not significantly improve the original results. The top 10 up- and downregulated DEGs were ranked after intervention of BQG compared with BLM model group, respectively (see Tables 4 and 5).

### 3.5. Notch Signaling Serves as a Candidate Pathway in Regulating SSc

**Figure 7:**
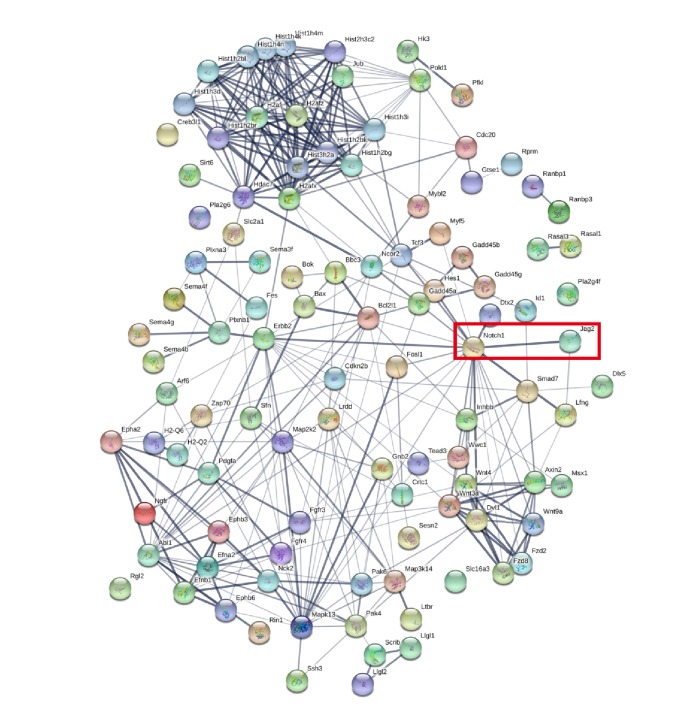
Genes were clustered in the PPI network according to string database (genes from top 10 predicted pathways in BQG-treated group versus BLM group).

In order to further discover the potential functional pathways variated by BQG, we carried out Kyoto Encyclopedia of Genes and Genomes (KEGG) enrichment analysis for DEGs, and the top 10 DEGs are listed in Table 6. Notch signaling pathway ranking 5 was priorly chosen to be verified in the following experiments (Figure 7). To explore the biological function of differentially expressed mRNA, Gene Ontology (GO) pathway annotation was conducted. It was revealed that, under the undistinguished circumstance of up- or downregulated relationship, single-organism cellular process was the most significantly enriched pathway for biological processes, compared with the BLM model group. In addition to the analysis of molecular function enrichment, the DEGs were enriched in protein binding. Moreover, intracellular part was the most obvious pathway of cellular component, which had the enriched DEG called Notch-1. Both up- and downregulated mRNAs significantly enriched GO terms, and the top 20 DEGs are presented in Table 7.

### 3.6. Notch-1 and Jagged-2 Play The Important Roles in The Pathological Progress of SSc

**Figure 8:**
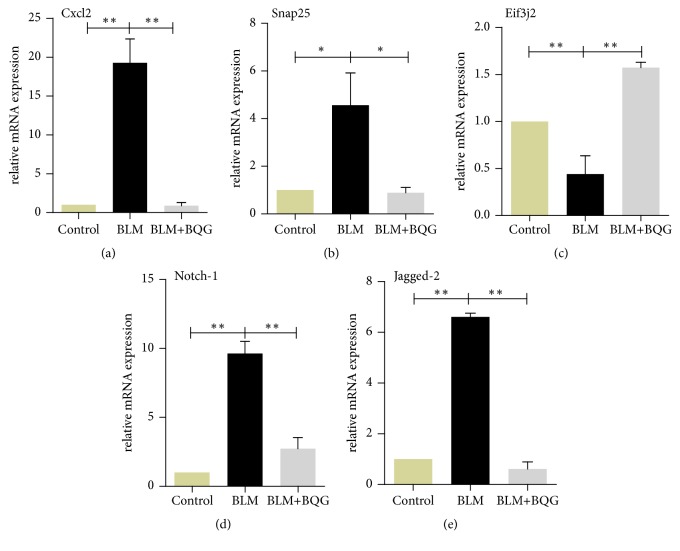
Validation of RT-qPCR. The predicted target genes, such as Cxcl2 (a), Snap25 (b), Eif3j2 (c), Notch-1 (d), and Jagged-2 (e), were selected for validation by RT-qPCR. Data were expressed as fold of changes compared to the control group from at least three biological replicates.

**Figure 9:**
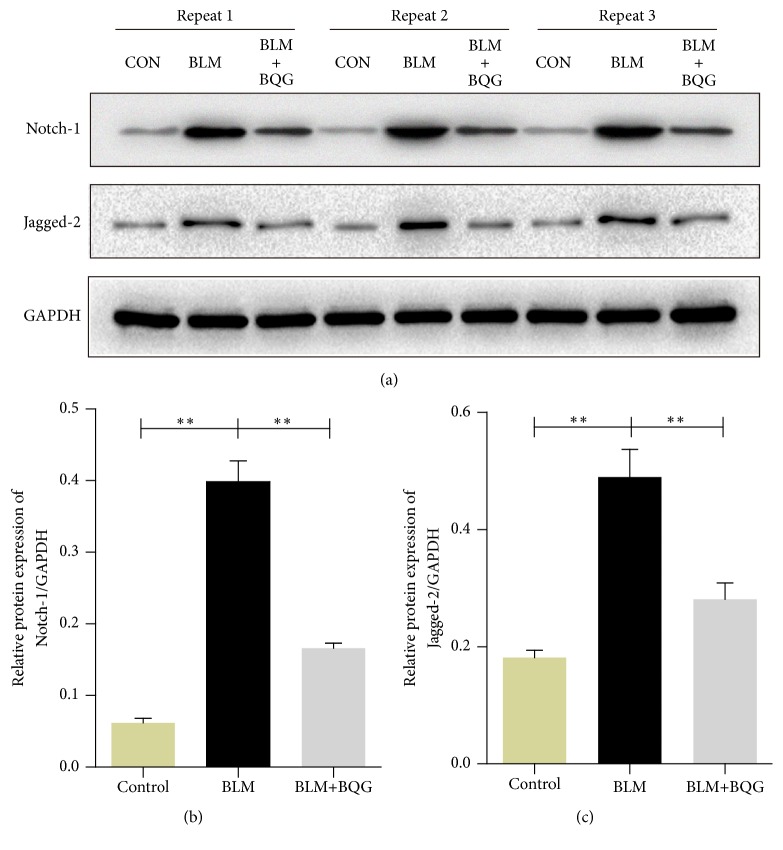
Validation of Western blotting. (a) Representative proteins of Notch signaling were detected exposed to BLM with or without BQG treatment in mice skin tissues. (b) Relative protein expression of Notch-1/GAPDH. (c) Relative protein expression of Jagged-2/GAPDH. ^∗^*P*<0.05, ^∗∗^*P*<0.01 compared with BLM model group.

Based on the above mentioned results, overlapped DEGs were identified as potential targets and further confirmed by RT-qPCR as well. In the results, the trends of Cxcl2, Synaptosomal-associated protein 25 (Snap25), Eukaryotic translation initiation factor 3, subunit J2 (Eif3j2), Notch-1, and Jagged-2 were illustrated in Figures 8(a)–8(e); thus, there was a statistical significance at the transcript expression level between the groups. In addition, Western blot analysis was carried out using the same remaining samples. It is noteworthy that Notch-1 and Jagged-2 (Figures 9(a)–9(c)) were significantly elevated in the BLM model group compared with the control group at the protein expression level. On the contrary, the decrease of the expression level of Notch-1 and Jagged-2 was detected in BQG-treated group. As a result, Notch signaling pathway was associated with proteins named Notch-1 and Jagged-2, which participated in the construction of SSc model and the treatment process of BQG.

## 4. Discussion

The purpose of our present study was to achieve the BQG's molecular evidence against dermal sclerosis out of mess and to provide a new evidence for network pharmacology. We showed the representative constitutes and the contents of BQG, and the BQG's preventive effects on SSc were revealed as well. Furthermore, 5 potential targets were sought out and validated in 1048 genes altered after BQG treatment. Thus, the possible mechanism was verified, which inhibited the Notch signaling pathway.

BQG, consisting of ten Chinese herbs, is mainly used for treating the SSc's “Lung deficiency generating phlegm stasis” syndrome. In addition, some evidences about its effects associated with fibrosis, which is the footstone of SSc, could be found in previous studies. Danshensu, one of the major components derived from* Salvia miltiorrhiza* Bunge, can attenuate cardiac fibrosis and hepatic fibrosis [22, 23]. Amygdalin can reduce the BLM-induced increase of proteinic biomarkers in rat serum [24]. It also can attenuate kidney fibroblast activation and rat renal interstitial fibrosis [25]. Paeonol has therapeutic functions on BLM-induced pulmonary fibrosis in mice and, at least in part, could be mediated by the inhibition of the MAPKs/Smad3 signaling pathway [26]. As the main active substance of* Astragalus membranaceus* Bunge, astragaloside IV also contains the potent protective effect on cardiovascular disease, pulmonary disease, and liver fibrosis [27]. Because BQG is a material basis for the treatment efficacy, the main focus in the next step will be to identify and separate precise active ingredients.

However, the effect of compound drugs cannot be replaced by a single component. To uncover the possible mechanism by a holistic approach, the BLM-induced SSc mouse model was previously tested successfully, which was consistent with our present in vivo study. Over time, BQG ameliorated the overall health of mice, including body weight. Thus, BQG-treated group attenuated the level of spleen index and increased thymus index conversely to a certain extent, indicating that BQG could prevent the occurrence of splenomegaly and play a protective role in immune system. Additionally, BQG administration stopped the tendency of skin sclerosis development reflected by not only pathological images, but also the HYP content. Cxcl2 is a chemokine primarily functions with recruiting neutrophils [28, 29]. Besides, we found that its expression level increased in model group, which was in agreement with the findings of a previous research [30]. BQG also significantly reduced Cxcl2 level, indicating that BQG alleviated inflammation partly due to Cxcl2 or the involvement of neutrophils. Vasculopathy characterized by *α*-SMA plays an important role in the pathogenesis of SSc [31, 32]. BQG was showed to be advantageous to improve the expression level of *α*-SMA-positive cells in vascular walls, and it was revealed that BQG's effect might be possibly related to vascular changes. Previous evidences supported this consequence as well [21, 31]. All these efforts provided a reliable sample basis for subsequent analysis. Additionally, we for the first time illustrated the underlying mechanisms of this old traditional formula using transcriptomics and bioinformatics analyses to examine the possible molecular targets affected by BQG. RNA-sequency expression profiling showed that 945 genes were altered after BQG administration. Three predicted target genes were selected for validation by RT-qPCR, whose fold-change tendencies of Cxcl2, Snap25, and Eif3j2 were consistent with transcriptomic data. These results consolidated that RNA-sequencing data were credible. Then, DEGs were analyzed by protein-protein interaction (PPI) network and Notch-1/Jagged-2 signaling was sort out. Furthermore, we confirmed them in gene level by RT-qPCR, and their associated proteins were validated by Western blotting as well.

**Figure 10:**
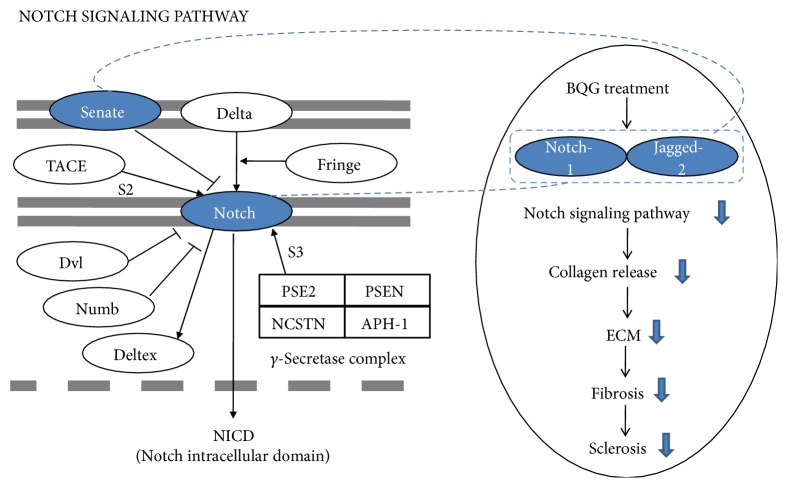
Schematic illustration of potential signaling pathways. Schematic diagram of BQG potential protective effects against skin fibrosis by regulating targets of Notch signaling pathway.

Notch signaling pathway ranked 5 in our data, and that is a conserved developmental pathway, participating in regulating all kinds of key cellular processes [33], as well as acting in the SSc pathogenesis. Clara et al. demonstrated that Notch signaling can be highly activated in SSc, and it also can promote collagen release and activation of fibroblast isolated from the skin samples. Additionally, fibrosis could be ameliorated by inhibition of Notch signaling pathway, applying a *γ*-secretase inhibitor (DAPT) or overexpression of a Notch-1 antisense construct [34, 35], which was in agreement with Kavian et al.'s findings [36]. It was reported that Notch deficiency resulted in a crucial inhibitory effect on the response to BLM-induced dermal fibrosis and lung fibrosis [37]. To our knowledge, core elements of this signaling pathway consists of four Notch transmembrane receptors and five transmembrane ligands in mammals named as follows: Notch 1-4, three Delta-like proteins (DLL1, DLL3, and DLL4), and two Jagged proteins (Jag 1, 2). However, the detailed contribution of the diverse Notch receptors has remained obscure, especially in terms of occurring in a variety of circumstances. Two-step proteolysis of the receptors was caused by binding and interaction between Notch receptors and their ligands. Then, an active form of the Notch intracellular domain (NICD) was released and translocated from cytoplasm to nucleus, where it ultimately interacted with transcriptional repressors, stimulating the expression level of various genes [38–40]. Several tissues express not only Jagged-2, but also Notch-1 because Notch-1 is a cognate receptor for Jagged-2 [41]. To date, there is no enough evidence on how Notch signaling pathway stimulates collagen release in fibrotic diseases on a molecular level. Here, as schematic diagram shown in Figure 10, we only provided a novel evidence that Notch-1 and Jagged-2 were also elevated in the BLM-induced SSc mouse model. Moreover, depending on BQG that repressed the expression level of Notch-1 and Jagged-2, it is suggested that the reduction of progress in skin sclerosis may be mediated by inhibition of the activation of Notch signaling pathway. Consequently, it could enhance the current understanding of Notch signal transduction in BLM-induced SSc mouse model, although it was not fully elucidated whether BQG acted directly or indirectly.

Nevertheless, some limitations of our study should be pointed out. Firstly, the presented Chinese medicinal formula is pretty complex, especially in the efficacy and mechanism of chemical components which needs to be further explored with more samples size. Secondly, as different mice models have different clinical features and molecular bases, more various mice models should be used to evaluate the effects of BQG on SSc treatment to further support our findings. Thirdly, a small part of target genes was confirmed in this experiment; the rest latently should be under consideration for the sake of moving as far as possible to “Precision Medicine” in Chinese herbs.

## 5. Conclusion

The results showed that the administration of BQG can prevent sclerotic skin induced by BLM in mice model, and this process is partly associated with decrease of inflammation, vascular changes, and fibrosis effects, so as to suppress the production of fibrillar collagens, which are modulated by blocking Notch signaling pathway.

## Figures and Tables

**Table 1:** The batch number and produced date of each herb.

Chinese name	Herb (Local name)	Medicinal parts	Amount (g)	Batch number
HangQi	Astragalus membranaceus (Fisch.) Bge.var. mongholicus (Bge.) Hsiao	Root	30 g	1705165
DangShen	Codonopsis pilosula (Franch.) Nannf.	Root	20 g	1706201
ShanYao	Dioscorea opposita Thunb.	rhizome	20 g	1705108
DanPi	Paeonia suffruticosaAndr.	root bark	12 g	17121101
DanShen	Salvia miltiorrhiza Bge.	Root	20 g	1706030
DangGui	Angelica sinensis (oliv.)Diels.	Root	10 g	1707182
TaoRen	Prunus persica(L) Batch.	Seed	10 g	17121371
WuWeizi	Schisandra chinensis(Turcz.) Baill.	Fruit	6 g	1706066
LingXiaoHua	Campsis grandiflora (Thunb.) K. Schum.	Flower	10 g	1609126
JieGeng	Platycodon grandiforum(Jacq) A. DC.	Root	6 g	18011431

**Table 2:** Primer sequences for RT-qPCR.

Targets	Forward primer (5'-3')	Reverse primer (3'-5')
Mus-Cxcl2	CCAACCACCAGGCTACAGG	GCGTCACACTCAAGCTCTG
Mus-Snap25	GCTGGAGGAGATGCAGAGGA	TCCAGTTGTTCGCCTTGCTC
Mus-Eif3j2	ACCAACTCATTGACTGTGCTCTGC	CCACCATAATCTGCCAGGTCGTC
Mus-Notch1	ATGCTGCTGTTGTGCTCCTGAAG	CGGCAATCGGTCCATGTGATCC
Mus-Jagged2	AAGGAGTACCAGGCCAAGGTGAC	CCGGCGGCAGGTAGAAGGAG
Mus-GAPDH	CACCATCTTCCAGGAGCGAG	CCTTCTCCATGGTGGTGAAGAC

**Table 3:** Identification of typical chemical components and its contents in BQG.

Peak	Identification	Content (ug/g)	Source
1	Amygdalin	2023.5	TaoRen
2	Paeonol	764.5	DanPi
3	Schisandrol A	38.7	WuWeiZi
4	Dihydrotanshinone I	9.3	DanShen
5	Cryptotanshinone	11.5	DanShen
6	Schizandrin A	0.7	WuWeiZi
7	Tanshinone IIA	9.9	DanShen
8	Schizandrin B	0.7	WuWeiZi
9	Danshensu	2290.2	DanShen
10	Acteoside	549.3	LingXiaoHua
11	Ferulic acid	45.2	DangGui
12	Lobetyolin	31.0	DangShen
13	Platycodin D	76.2	JieGeng
14	Astragaloside IV	196.0	HangQi

**Table 4:** Upregulated DEGs after intervention of BQG.

Gene ID	Gene Name	log2 fold change	P-value
ENSMUSG00000097656	Gm26712	7.98659314	1.31E-06
ENSMUSG00000105338	Gm43802	7.80359975	3.28E-08
ENSMUSG00000106587	AC125099.2	7.470051892	3.57E-06
ENSMUSG00000103308	Gm37800	4.023628692	4.11E-05
ENSMUSG00000049421	Zfp260	3.689570886	9.22E-06
ENSMUSG00000003545	Fosb	3.68790389	3.35E-06
ENSMUSG00000057465	Saa2	3.572774437	2.45E-06
ENSMUSG00000079025	Gsdmc	3.355515254	8.02E-06
ENSMUSG00000043424	Eif3j2	3.102051328	2.66E-07
ENSMUSG00000046311	Zfp62	2.135549165	1.47E-06

**Table 5:** Downregulated DEGs after intervention of BQG.

Gene ID	Gene Name	log2 fold change	P-value
ENSMUSG00000027273	Snap25	-6.26729	1.36E-05
ENSMUSG00000084257	Gm11597	-6.26702	8.33E-05
ENSMUSG00000083405	Gm15725	-5.90441	6.95E-07
ENSMUSG00000102564	Gm37035	-5.4123	6.22E-08
ENSMUSG00000058427	Cxcl2	-5.30387	4.51E-06
ENSMUSG00000097724	Gm26850	-5.06965	1.66E-05
ENSMUSG00000099068	Gm27861	-4.83004	4.91E-05
ENSMUSG00000029379	Cxcl3	-4.54472	8.15E-05
ENSMUSG00000097078	Gm26566	-4.25859	9.63E-05
ENSMUSG00000103309	BC037039	-4.22422	3.50E-05

**Table 6:** Signaling pathway prediction of DEGs between BQG and BLM group from KEGG database.

ID	Description	P-value	Significant symbol genes
UP-REGULATED			

ko03010	Ribosome	0.001525	Rpl30, Rpl26, Rpl35a, Rpl31, Rps27a, Rpl27-ps3
ko04924	Renin secretion	0.002328	Calm4, Gucy1a3, Adcyap1r1, Gucy1b3
ko03030	DNA replication	0.004173	Rnaseh2b, Rpa3, Mcm6
ko04624	Toll and Imd signaling pathway	0.004522	Birc3, Tab2, Faf1
ko04713	Circadian entrainment	0.008117	Calm4, Gucy1a3, Adcyap1r1, Gucy1b3
ko05031	Amphetamine addiction	0.018481	Calm4, Fosb, Ppp1cb
ko00230	Purine metabolism	0.018499	Nt5e, Entpd5, Gucy1a3, Gucy1b3, Pde4d
ko04970	Salivary secretion	0.020157	Calm4, Gucy1a3, Gucy1b3
ko04611	Platelet activation	0.022505	Gucy1a3, Vamp8, Ppp1cb, Gucy1b3
ko04270	Vascular smooth muscle contraction	0.023148	Calm4, Gucy1a3, Ppp1cb, Gucy1b3

DOWN-REGULATED			

ko04550	Signaling pathways regulating pluripotency of stem cells	0.001454	Inhbb, Wnt4, Map2k2, Dvl1, Mapk13, Id1, Fgfr3, Axin2, Tcf3, Myf5, Wnt9a, Fzd8, Fzd2, Dlx5, Wnt3a, Fgfr4
ko04360	Axon guidance	0.002752	Wnt4, Sema3f, Sema4g, Efnb1, Plxna3, Ssh3, Epha2, Pak6, Abl1, Pak4, Ephb3, Plxnb1, Efna2, Fes, Nck2, Ephb6, Sema4f, Sema4b
ko04115	p53 signaling pathway	0.002898	Sfn, Gtse1, Gadd45g, Gadd45a, Rprm, Gadd45b, Pidd1, Sesn2, Bbc3, Bax
ko04390	Hippo signaling pathway	0.003971	Wnt4, Dvl1, Llgl2, Id1, Axin2, Llgl1, Wnt9a, Fzd8, Fzd2, Wwc1, Scrib, Tead3, Ajuba, Wnt3a, Bbc3, Smad7
ko04330	Notch signaling pathway	0.006239	Dvl1, Hes1, Dtx2, Lfng, Jag2, Notch1, Ncor2
ko05166	HTLV-I infection	0.006668	Wnt4, Dvl1, Cdc20, Ranbp3, Ranbp1, Crtc1, Map3k14, Tcf3, Pold1, H2-Q2, Wnt9a, Msx1, Bcl2l1, Fzd8, Fzd2, Cdkn2b, H2-Q6, Ltbr, Wnt3a, Pdgfa, Slc2a1, Mybl2, Bax, Fosl1
ko04014	Ras signaling pathway	0.010254	Ngfr, Map2k2, Pla2g6, Rgl2, Arf6, Gnb2, Fgfr3, Rin1, Epha2, Pak6, Abl1, Pak4, Rasal3, Pla2g4f, Efna2, Bcl2l1, Zap70, Pdgfa, Fgfr4, Rasal1
ko05322	Systemic lupus erythematosus	0.012114	Hist1h2bl, Hist3h2a, Hist1h2br, Hist1h4n, Hist1h4m, H2afz, Hist1h3d, H2afj, Hist1h4k, Hist2h3c2, Hist1h3i, Hist1h2bg, Hist1h2bk, H2afx
ko00680	Methane metabolism	0.012152	Shmt2, Eno1b, Pfkl, Phgdh, Eno1
ko05230	Central carbon metabolism in cancer	0.014355	Slc16a3, Map2k2, Fgfr3, Hk3, Pfkl, Sirt6, Erbb2, Slc2a1

**Table 7:** The significantly enriched GO terms of DEGs ranking top 20.

Ontology	ID	Term	Gene count	P-value
UP-REGULATED				

biological process	GO: 0008152	metabolic process	119	4.60E-07
	GO: 0044237	cellular metabolic process	107	4.20E-06
	GO: 0044238	primary metabolic process	108	6.30E-06
	GO: 0071704	organic substance metabolic process	111	6.90E-06
molecular function	GO: 0003735	structural constituent of ribosome	11	5.00E-07
	GO: 0035662	Toll-like receptor 4 binding	3	5.10E-06
cellular component	GO: 0044445	cytosolic part	12	1.20E-06
	GO: 0022626	cytosolic ribosome	9	1.60E-06
	GO: 0022625	cytosolic large ribosomal subunit	7	4.10E-06
	GO: 0044391	ribosomal subunit	10	9.50E-06
	GO: 0005840	ribosome	11	1.30E-05
	GO: 0005683	U7 snRNP	3	2.90E-05
	GO: 0005687	U4 snRNP	3	8.40E-05
	GO: 0005615	extracellular space	29	0.00012
	GO: 0015934	large ribosomal subunit	7	0.00014
	GO: 0005576	extracellular region	58	0.00016
	GO: 0032991	macromolecular complex	61	0.00016
	GO: 0034709	methylosome	3	0.00018
	GO: 0044421	extracellular region part	52	0.00023
	GO: 0034719	SMN-Sm protein complex	3	0.00033

DOWN-REGULATED				

cellular component	GO: 0044424	intracellular part	839	3.60E-30
	GO: 0005622	intracellular	858	1.50E-27
	GO: 0043229	intracellular organelle	737	6.00E-22
	GO: 0043226	organelle	787	1.90E-21
	GO: 0005737	cytoplasm	660	1.30E-19
	GO: 0043227	membrane-bounded organelle	724	7.80E-18
	GO: 0044446	intracellular organelle part	511	1.30E-15
	GO: 0043231	intracellular membrane-bounded organelle	630	2.10E-15
	GO: 0044422	organelle part	519	5.80E-15
molecular function	GO: 0005515	protein binding	567	9.00E-22
biological process	GO: 0044763	single-organism cellular process	641	2.60E-18
	GO: 0007275	multicellular organism development	364	2.70E-17
	GO: 0048731	system development	333	5.90E-17
	GO: 0009888	tissue development	166	2.00E-16
	GO: 0048518	positive regulation of biological process	379	2.50E-15
	GO: 0048513	animal organ development	260	3.70E-15
	GO: 0048856	anatomical structure development	383	3.90E-15
	GO: 0043588	skin development	48	4.90E-15
	GO: 0044707	single-multicellular organism process	407	5.00E-15
	GO: 0032502	developmental process	407	1.10E-14

## Data Availability

The data used to support the findings of this study are available from the corresponding author upon request.

## Abbreviations

BLM: Bleomycin
BQG: Bufei Qingyu Granule
Cxcl2: Chemokine (C-X-C motif) ligand 2
DEGs: Differentially expressed genes
Eif3j2: Eukaryotic translation initiation factor 3, subunit J2
ELISA: Enzyme-Linked Immunosorbent Assay
ESI: Electrospray ionization
GO: Gene Ontology
H&E: Hematoxylin and eosin
HYP: Hydroxyproline
KEGG: Kyoto Encyclopedia of Genes and Genomes
NICD: Notch intracellular domain
PBS: Phosphate buffered saline
PVDF: Polyvinylidene difluoride
RIPA: Radio immunoprecipitation assay
SD: Standard deviation
Snap25: Synaptosomal-associated protein 25
SSc: Systemic sclerosis
TCM: Traditional Chinese medicine
TPM: Transcripts Per Million
UHPLC-Q-TOF/MS: Ultraperformance liquid chromatography coupled with quadrupole-time-of-flight mass spectrometry
*α*-SMA: *α*-smooth muscle actin.

## References

[1] Affandi A. J., Radstake T. R. D. J., Marut W. (2015). Update on biomarkers in systemic sclerosis: tools for diagnosis and treatment. *Seminars in Immunopathology*.

[2] Denton C. P., Khanna D. (2017). Systemic sclerosis. *The Lancet*.

[3] Bhattacharyya S., Wei J., Tourtellotte W. G., Hinchcliff M., Gottardi C. G., Varga J. (2012). Fibrosis in systemic sclerosis: common and unique pathobiology. *Fibrogenesis & Tissue Repair*.

[4] Taniguchi T., Asano Y., Akamata K. (2015). Fibrosis, vascular activation, and immune abnormalities resembling systemic sclerosis in bleomycin-treated Fli-1-haploinsufficient mice. *Arthritis & Rheumatology*.

[5] Dowson C., Simpson N., Duffy L., O’Reilly S. (2017). Innate immunity in systemic sclerosis. *Current Rheumatology Reports*.

[6] Distler J. H. W., Feghali-Bostwick C., Soare A., Asano Y., Distler O., Abraham D. J. (2017). Review: frontiers of antifibrotic therapy in systemic sclerosis. *Arthritis & Rheumatology*.

[7] Huang P. P., Wang S. G., Hua G. X. (1994). Observation on blood flow changes in 34 cases of progressive systemic scleroderma treated with Chinese herbal medicine. *Chinese Journal of Integrated Traditional and Western Medicine*.

[8] Wu T., Chu H., Tu W. (2014). Dissection of the mechanism of traditional Chinese medical prescription-Yiqihuoxue formula as an effective anti-fibrotic treatment for systemic sclerosis. *BMC Complementary and Alternative Medicine*.

[9] Han L., Bian H., Ouyang J., Bi Y., Yang L., Ye S. (2016). Wenyang Huazhuo Tongluo formula, a Chinese herbal decoction, improves skin fibrosis by promoting apoptosis and inhibiting proliferation through down-regulation of survivin and cyclin D1 in systemic sclerosis. *BMC Complementary and Alternative Medicine*.

[10] Zhao J.-R., Qu L., Li X.-M. (2004). Preventive and therapeutic effects of astragalus and angelica mixture on renal tubulointerstitial fibrosis after unilateral ureteral obstruction in rats. *Journal of Peking University Health sciences*.

[11] Wang H., Li J., Yu L., Zhao Y., Ding W. (2004). Antifibrotic effect of the Chinese herbs, Astragalus mongholicus and Angelica sinensis, in a rat model of chronic puromycin aminonucleoside nephrosis. *Life Sciences*.

[12] He S., Yang Y., Liu X. (2012). Compound Astragalus and Salvia miltiorrhiza extract inhibits cell proliferation, invasion and collagen synthesis in keloid fibroblasts by mediating transforming growth factor-*β*/Smad pathway. *British Journal of Dermatology*.

[13] Yang Y., Yang S., Chen M., Zhang X., Zou Y., Zhang X. (2008). Compound astragalus and salvia miltiorrhiza extract exerts anti-fibrosis by mediating TGF-*β*/Smad signaling in myofibroblasts. *Journal of Ethnopharmacology*.

[14] Wu C., Jiang J., Boye A., Jiang Y., Yang Y. (2014). Compound astragalus and salvia miltiorrhiza extract suppresses rabbits' hypertrophic scar by modulating the TGF-*β*/smad signal. *Dermatology*.

[15] Qian X., Zhu X.-X., Chen X.-Y. (2006). Effect of Bufei Qingyu Granule in mollifying skin of mouse scleroderma model. *Chinese Journal of Integrated Traditional and Western Medicine*.

[16] Jiang L., Li X., Zhang Y., Zhang M., Tang Z., Lv K. (2017). Microarray and bioinformatics analyses of gene expression profiles in BALB/c murine macrophage polarization. *Molecular Medicine Reports*.

[17] Wang H., Xing W., Tang S. (2017). Du formula alleviates diabetic retinopathy in rats by inhibiting SOCS3-STAT3 and TIMP1-A2M pathways. *International Journal of Genomics*.

[18] Avouac J. (2014). Mouse model of experimental dermal fibrosis: The bleomycin-induced dermal fibrosis. *Methods in Molecular Biology*.

[19] Yamashita T., Asano Y., Taniguchi T. (2017). Glycyrrhizin ameliorates fibrosis, vasculopathy, and inflammation in animal models of systemic sclerosis. *Journal of Investigative Dermatology*.

[20] Morin F., Kavian N., Chouzenoux S. (2017). Leflunomide prevents ROS-induced systemic fibrosis in mice. *Free Radical Biology & Medicine*.

[21] Qi Q., Mao Y., Tian Y. (2017). Geniposide inhibited endothelial-mesenchymal transition via the mTOR signaling pathway in a bleomycin-induced scleroderma mouse model. *American Journal of Translational Research*.

[22] Lu H., Tian A., Wu J., Yang C., Xing R., Jia P. (2014). Danshensu inhibits beta-adrenergic receptors-mediated cardiac fibrosis by ROS/p38 MAPK axis. *Biological & Pharmaceutical Bulletin*.

[23] Qu W., Huang H., Li K., Qin C. (2014). Danshensu-mediated protective effect against hepatic fibrosis induced by carbon tetrachloride in rats. *Pathologie Biologie*.

[24] Du H.-K., Song F.-C., Zhou X., Li H., Zhang J.-P. (2010). Effect of amygdalin on serum proteinic biomarker in pulmonary fibrosis of bleomycin-induced rat. *Chinese Journal of Industrial Hygiene And Occupational Diseases*.

[25] Guo J., Wu W., Sheng M., Yang S., Tan J. (2013). Amygdalin inhibits renal fibrosis in chronic kidney disease. *Molecular Medicine Reports*.

[26] Liu M.-H., Lin A.-H., Ko H.-K., Perng D.-W., Lee T.-S., Kou Y. R. (2017). Prevention of bleomycin-induced pulmonary inflammation and fibrosis in mice by paeonol. *Frontiers in Physiology*.

[27] Li L., Hou X., Xu R., Liu C., Tu M. (2017). Research review on the pharmacological effects of astragaloside IV. *Fundamental & Clinical Pharmacology*.

[28] Marcos V., Zhou Z., Yildirim A. Ö. (2011). CXCR2 mediates NADPH oxidase-independent neutrophil extracellular trap formation in cystic fibrosis airway inflammation. *Nature Medicine*.

[29] De Filippo K., Dudeck A., Hasenberg M. (2013). Mast cell and macrophage chemokines CXCL1/CXCL2 control the early stage of neutrophil recruitment during tissue inflammation. *Blood*.

[30] Liang M., Lv J., Zou L. (2015). A modified murine model of systemic sclerosis: Bleomycin given by pump infusion induced skin and pulmonary inflammation and fibrosis. *Laboratory Investigation*.

[31] Yamamoto T., Katayama I. (2011). Vascular changes in bleomycin-induced scleroderma. *International Journal of Rheumatology*.

[32] Trojanowska M. (2010). Cellular and molecular aspects of vascular dysfunction in systemic sclerosis. *Nature Reviews Rheumatology*.

[33] Sasnauskiene A., Jonusiene V., Krikstaponiene A. (2014). NOTCH1, NOTCH3, NOTCH4, and JAG2 protein levels in human endometrial cancer. *Medicina (Lithuania)*.

[34] Dees C., Zerr P., Tomcik M. (2011). Inhibition of Notch signaling prevents experimental fibrosis and induces regression of established fibrosis. *Arthritis & Rheumatology*.

[35] Dees C., Tomcik M., Zerr P. (2011). Notch signalling regulates fibroblast activation and collagen release in systemic sclerosis. *Annals of the Rheumatic Diseases*.

[36] Kavian N., Servettaz A., Mongaret C. (2010). Targeting ADAM-17/notch signaling abrogates the development of systemic sclerosis in a murine model. *Arthritis & Rheumatology*.

[37] Liu Y., Huang G., Mo B., Wang C. (2017). Artesunate ameliorates lung fibrosis via inhibiting the notch signaling pathway. *Experimental and Therapeutic Medicine*.

[38] Zong D., Ouyang R., Li J., Chen Y., Chen P. (2016). Notch signaling in lung diseases: Focus on Notch1 and Notch3. *Therapeutic Advances in Respiratory Disease*.

[39] Hu B., Phan S. H. (2016). Notch in fibrosis and as a target of anti-fibrotic therapy. *Pharmacological Research*.

[40] Nam D.-H., Jeon H.-M., Kim S. (2008). Activation of Notch signaling in a xenograft model of brain metastasis. *Clinical Cancer Research*.

[41] Luo B., Aster J. C., Hasserjian R. P., Kuo F., Sklar J. (1997). Isolation and functional analysis of a cDNA for human Jagged2, a gene encoding a ligand for the Notch1 receptor. *Molecular and Cellular Biology*.

